# Maturation and Post-Harvest Resting of Fruits Affect the Macronutrients and Protein Content in Sweet Pepper Seeds

**DOI:** 10.3390/plants11162084

**Published:** 2022-08-10

**Authors:** Lidiane Fernandes Colombari, Larissa Chamma, Gustavo Ferreira da Silva, Willian Aparecido Leoti Zanetti, Fernando Ferrari Putti, Antonio Ismael Inácio Cardoso

**Affiliations:** 1 Department of Crop Science, School of Agriculture, São Paulo State University (UNESP), Botucatu 18610-034, Brazil; 2 Department of Biosystems Engineering, School of Sciences and Engineering, São Paulo State University (UNESP), Tupã 17602-496, Brazil

**Keywords:** *Capsicum annuum* L., maturation, nutritional quality, seed chemical composition

## Abstract

There are few studies about the influence of fruit maturation and post-harvest resting on seed composition, which can be necessary for seedling development and future establishment. Thus, the objective of this study was to evaluate the effect of maturation and post-harvest resting of fruits on the macronutrient and protein content of sweet pepper seeds. The experimental design was a randomized block, with eight treatments, in a 4 × 2 factorial arrangement. The first factor was fruit maturation stages (35, 50, 65 and 80 days after anthesis), and the second, with and without post-harvest resting of the fruits for 7 days. The characteristics evaluated in seeds were the dry weight of one thousand seeds, macronutrient content, and content of albumin, globulin, prolamin and glutelin proteins. There were reductions in K, Ca and Mg content, and an increase in seed content of albumin, globulin and prolamins as a function of the fruit maturation stage. Post-harvest resting of the fruits provided higher Ca content and protein albumin in seeds. The decreasing order of macronutrients and protein content in seeds, independent of fruit maturation and resting stage of the fruits, was N > K > P > Mg > S > Ca, and albumin > globulin ≈ glutelin > prolamine, respectively.

## 1. Introduction

The main compounds that seeds store are carbohydrates, lipids, proteins, minerals, vitamins and plant hormones [1]. During plant development, nutrients are translocated to the fruits, and later, to seeds. The nutritional requirement of plants becomes intense in the reproductive phase, being more critical during seed formation, where the stored compounds will influence the formation of the embryo, and consequently, the metabolism, vigor and storage capacity of seeds [2,3]. Nitrogen is the most accumulated nutrient in seeds, followed by potassium and phosphorus, but it depends on the species [4,5]. As for protein reserves, two classes are mainly found in seeds: albumins and globulins, or prolamins [6].

Seed proteins are the main sources of nitrogen and sulfur, indispensable for the synthesis of new proteins, nucleic acids and secondary compounds [6,7,8]. Subsequently, the proteins that were synthesized and stored in the seeds will be broken down into amino acids for biosynthesis and energy generation, and, together with the other reserves, will be mobilized during germination for the development of the embryo, until the seedling manages to emerge above the ground and becomes photosynthetically active [6,9,10].

In the production of sweet pepper seeds, the fruits must be harvested at physiological maturity, when the maximum accumulation of dry matter occurs. If harvested before this point, the fruits must remain at rest after harvesting before extracting seeds for seven to ten days. This procedure allows early harvesting, reducing the time the fruits are exposed to unfavorable climatic conditions and the attack of pests and diseases [11,12]. During the post-harvest resting, the complete formation of the biochemical, morphological, and structural systems of seeds occurs [12,13].

During the maturation period and fruit post-harvest resting, the weight of seeds increases [11,12], probably because of translocation and accumulation of reserve compounds in seeds. However, there is no research showing the changes of this species in the chemical composition of seeds at each stage of fruit maturation and during fruit rest after harvest. Knowledge of these factors can help identify the best fertilization management approach, as it can indicate which nutrients are most important at each stage of plant development. Thus, the objective of this study was to evaluate the effect of maturation and post-harvest resting of fruits on the macronutrient and protein content of sweet pepper seeds.

## 2. Results

### 2.1. Water Content, Dry Weight of One Thousand and Macronutrient Content of Seeds

There was only a significant interaction between factors (maturation periods and post-harvest resting of the fruits) for nitrogen and sulfur content in the seeds. Therefore, for all other parameters, the factors were analyzed separately (Table 1 and Table 2).

**Figure 1:**
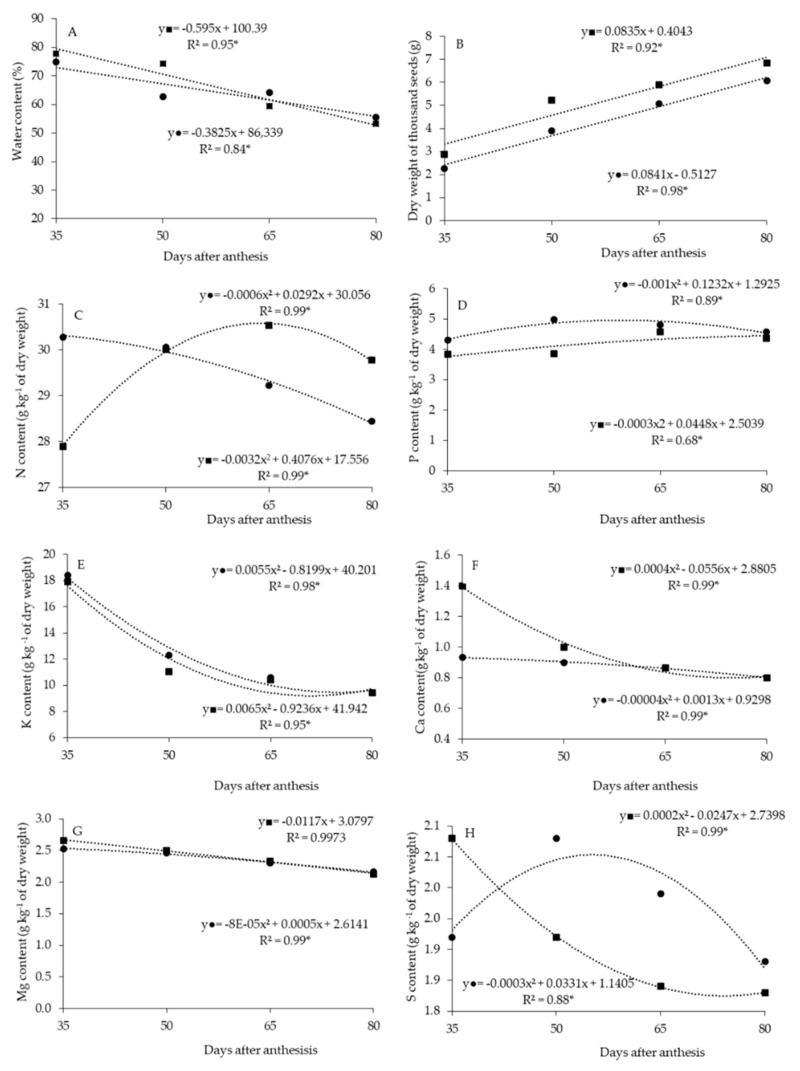
Seed water content (**A**), dry weight of one thousand seeds (**B**), nitrogen—N (**C**), phosphorus—P (**D**), potassium—K (**E**), calcium—Ca (**F**), magnesium—Mg (**G**) and sulfur—S (**H**) content in sweet pepper seeds, as a function of maturation stage, without (●) and with (■) post-harvest resting of fruits.* significant difference at 5% probability.

Seed water content was adjusted to the decreasing linear model as a function of the maturation stage. The highest values were obtained at 35 DAA, and they reduced over time to a minimum of 56% and 53% at 80 DAA, without and with post-harvest fruit resting, respectively (Figure 1A).

A linear increase in dry weight of one thousand seeds (DWTS) was observed in fruit maturation, reaching a maximum value of 6.6 and 7.7 g at 80 DAA in seeds without and with post-harvest resting, respectively (Figure 1B).

There was a reduction of N content in seeds without fruit rest as a function of the maturation stage, with a minimum content of 28.5 g kg^−1^ of dry matter (DM) at 80 DAA (Figure 1C). The N content had a quadratic response with fruit rest, with a maximum estimated at 30.5 g kg^−1^ of DM, at 64 DAA.

Seed P contents were adjusted to the quadratic model, being estimated at a maximum of 5.1 g kg^−1^ of DM at 62 DAA, without rest, and 4.2 g kg^−1^ of DM at 74 DAA, with post-harvest resting of the fruits (Figure 1D).

For K, Ca and Mg contents in the seeds, there were reductions during most periods of maturation, obtaining minimum contents of 9.6, 0.81 and 2.2 g kg^−1^ of DM at 74, 80 and 80 DAA, respectively, without post-harvest resting. For fruits with post-harvest resting, the minimum contents were 9.1, 0.95 and 2.1 g kg^−1^ of DM at 71, 70 and 80 DAA for K, Ca and Mg, respectively (Figure 1E–G). 

S contents in seeds were also adjusted to the quadratic model; however, without fruit rest, there was an increase up to 50 DAA, with a reduction after this date. On the other hand, there was a reduction in contents up to 65 DAA with post-harvest resting, and a slight increase in values after this period (Figure 1H).

Comparing the factors post-harvest resting of the fruits, the presence of rest for seven days provided higher DWTS (Table 1). The post-harvest resting of the fruits also provided higher Ca content. Conversely, it was observed that the P content was higher without rest (Table 1).

Seed N content at 35 DAA was higher without fruit rest; however, at 65 and 80 DAA, higher contents were obtained after rest (Table 2). Without post-harvest resting, the N content in seeds reduced linearly with fruit maturation (Figure 1C); with rest, there was an increase until 64 DAA, and after this, a small decrease was observed.

The S content of seeds at 35 DAA was higher with fruit rest, but at 50 and 65 DAA, the contents were higher without fruit rest (Table 2). 

Nutrient contents in the sweet pepper seeds followed the following decreasing order: N > K > P > Mg > S > Ca.

### 2.2. Seed Protein Content

**Figure 2:**
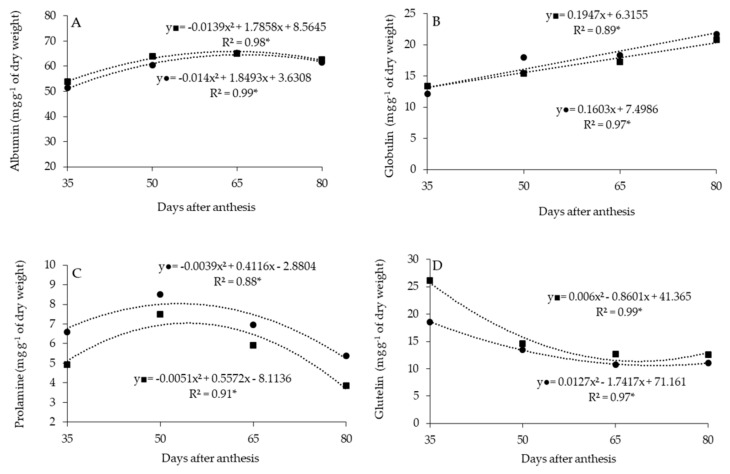
Albumin (**A**), globulin (**B**), prolamine (**C**) and glutelin (**D**) content in sweet pepper seeds, as a function of maturation stage, without (●) and with (■) post-harvest resting of fruits. * significant difference at 5% probability.

For proteins, the content of albumin and prolamine had quadratic responses, with maximum estimated values without fruit rest of 64.7 and 7.9 mg g^−1^ of DM, at 66 and 53 DAA, respectively. Post-harvest resting, the maximum albumin content was estimated at 65.9 mg g^−1^ of DM at 64 DAA, and prolamine was estimated at 7.1 mg g^−1^ of DM at 55 DAA (Figure 2A,C).

Globulin protein had a linear increase, with maximum contents of 21.9 and 20.3 mg g^−1^ of DM, at 80 DAA, without and with fruit rest, respectively (Figure 2B).

Glutelin content adjusted to the quadratic model according to the maturation stage; however, there were reductions over the maturation stage, with the minimum without fruit rest estimated at 10.5 mg g^−1^ of DM at 72 DAA, and with rest estimated at 11.4 mg g^−1^ of DM at 68 DAA (Figure 2D).

It was observed that the post-harvest resting of the fruits enabled higher contents of albumin and glutelin. However, the prolamine protein content was higher without fruit resting (Table 3). Protein content in the sweet pepper seeds followed the following decreasing order: albumin > globulin ≈ glutelin > prolamine.

## 3. Discussion

### 3.1. Water Content, Dry Weight of One Thousand and Macronutrients Content of Seeds

During maturation inside the fruits, seeds maintain a high water content (35 to 40%) and undergo dry matter accumulation. Water is considered the vehicle for photoassimilate translocation from the plant to the seeds, so it is necessary to synthesize their reserves [6,14]. However, the water content necessary is lower than that needed to initiate germination [11,12]. 

Studies conducted by refs. [13,14] determined the minimum water content in ‘Magda’ sweet pepper seeds to be 54% at 70 DAA, and 47% at 75 DAA in sweet yellow pepper, without fruit rest. The reduction in seed water content as a function of the fruit maturation stage was also reported in other species of the *Capsicum* genus [15,16].

An increase in the dry weight of one thousand seeds (DWTS) occurred because seeds tend to increase the dry weight until physiological maturity during the maturation process [17]. 

The beginning of seed development was characterized by the relatively slow accumulation of dry mass. This phase predominates the cell division and expansion, which are responsible for the constitution of the adequate structure to receive the substances transferred from the mother plant. Soon after, the replacement of water content with dry matter begins after the initial seed growth [17,18].

The stage of fruit harvest for better physiological seed quality can change according to species, cultivar and environmental conditions [13,19,20,21,22]. For the DWTS, higher values were obtained after post-harvest resting. Similar results were reported in other pepper seeds [23,24,25]. 

The post-harvest temporary storage of fruits before extraction allows the seeds to complete their physiological maturation [12,13,26,27]. Thus, the reserves continue to be metabolized and translocated to the seeds, allowing increases in weight and improving the physiological and nutritional quality of the seed.

Post-harvest resting of fruits is especially important in species with an indeterminate growth habit that produce fleshy fruits, such as pepper, cucumber, tomato and other species. The post-harvest resting in species with indeterminate growth habits is helpful to improve the uniformity generated by continuous flowering, reducing the number of harvests and the exposure of fruits and seeds to unfavorable field conditions [24].

An increase in the DWTS during the maturation stage is associated with the amount of reserve accumulated during seed maturation. Studies of the sweet pepper cultivar Amarela Comprida demonstrated increases in DWTS up to 75 DAA, with a maximum weight of 6.6 g [28]. However, in the pepper cultivar ‘Malagueta’, the maximum DWTS obtained was 3.2 g at 80 DAA, and in the ‘Biquinho’ pepper, it was 2.5 g at 70 DAA [25], thus showing that values can vary according to genotype.

The decreasing order of macronutrient content in the seeds was N > K > P > Mg > S > Ca. N was the most accumulated in sweet pepper seeds. Several studies reported that the N content in the seeds was always higher than the other nutrients [4,5,29,30]. Nitrogen occupies a prominent place in the plant metabolism system because all vital plant processes are associated with proteins, in which N is an essential constituent [31,32].

N is one of the most easily translocated nutrients from leaves to fruits [33]. However, there are no studies of this translocation from fruits at rest to seeds, as may have occurred in this research, mainly in older fruits (more than 65 DAA). In younger fruits, with rest, there may have been a “dilution effect”; that is, the increase in dry seed weight with rest may have been greater than the translocation of N to seeds. In comparison, in older fruits, it is possible that the N translocation rate from fruit to seeds was more intense, favoring the increase of N content (Figure 1C and Table 2). “Nutrient dilution” is characterized when the DM growth rate is higher than the nutrient absorption rate. Similarly, it can occur in the seeds. With the advance of the maturation stage, together with the rest of the fruits, it allows the continuity of the seed maturation process and DM accumulation.

P was the third most accumulated nutrient in seeds. Phosphorous compounds are important in several reactions observed in seeds [34]. Furthermore, phosphorus is a constituent of the nucleic acid molecule, related to protein synthesis. Additionally, P is present in phospholipids and phosphate sugars, nucleotides and phytin, which is a salt with calcium and magnesium in seeds [30,35]. The requirement of this macronutrient for seeds may be associated with the fact that this nutrient provides faster initial root growth, improving initial seedling establishment. Seeds usually contain enough P to ensure maximum seedling growth for several weeks after germination [36].

The content of P in seeds is probably regulated by plants so that there is no deficiency of this nutrient during seed germination and the beginning of plant development. When there is a lack of P in the soil, it is translocated from the leaves to the fruits and seeds [37].

Regarding K, Ca and Mg content, the behavior was different from the dry matter accumulation in seeds. The content of these macronutrients had a reduction during most periods of the maturation stage (Figure 1E–G). According to [38], as the plant grows, nutrients are diluted, reducing the concentration in the tissues. The same probably happens in seeds as the maturation stages advance.

K was the second most accumulated nutrient in seeds, confirming the importance of this element in seed formation. The physiological role of K during fruit formation and maturation is mainly expressed in carbohydrate metabolism [39], which also makes it one of the most accumulated nutrients of sweet pepper seeds.

Ca was the nutrient with the lowest content in sweet pepper seeds, probably due to its low mobility in the phloem. According to ref. [29], Ca accumulation in seeds occurs only by absorption and transport during their maturation process, with no redistribution. Therefore, although fruit resting increases seed DM, it does not provide continuity of translocation of all macronutrients. This translocation occurs at a lower intensity than DM accumulation and is called the “dilution effect”.

Mg in seeds is associated with proteins. However, its role is not yet fully understood, whether related to protein formation or whether this is a consequence of increased amino acid translocation from leaves to drains [40].

Without post-harvest resting, S content during maturation stages increased until 50 DAA, and decreased in older fruits (Figure 1H and Table 2), similarly to N, probably because of the “dilution effect”, when the DM growth rate is higher than the nutrient absorption rate. With post-harvest resting, there is DM accumulation due to the maturation stage and to resting; as such, the S content decreases during all maturation stages, similarly to most nutrients. S regulates seed metabolism in terms of carbohydrates and storage proteins. S content in sweet pepper seeds was higher than Ca; S is responsible for carbohydrate regulation and storage of protein seed metabolism [41]. It is essential to mention that the S content in the seeds is low in most species, and that the mineral composition of the seeds can vary according to the species [42], emphasizing the importance of studying the mineral composition of seeds in different species. However, in cauliflower (*Brassica oleracea*), S was the second most accumulated nutrient in the seeds [30].

### 3.2. Seed Protein Content

Although proteins are part of seed reserves, not all groups are found in the seeds of a determined species. Albumin and globulin are standard in dicotyledonous seeds [8]. They are probably translocated in greater proportions to sweet pepper seeds, favoring their concentration, with albumin, the main storage protein, being present in greater quantity (Figure 2). The present study observed an increase in albumin content during maturation stages (Figure 2A), and, according to ref. [43], there are relationships between protein content, especially albumin, and seed physiological quality.

Glutelins are common proteins in cereals, and prolamins are common in grasses [8]; this may justify the lower prolamine content in sweet pepper seeds. Thus, plants accumulate reserves such as carbohydrates, oils and proteins during the maturation process, which are essential for germination and establishment [44]. As such, at the beginning of the germination process, the seeds are soaked in water, which begins the mobilization of a food reserve. The storage organs (cotyledons and endosperm) provide essential energy to nourish the seedling until stabilization [45]. Thus, the present result obtained in the study corroborates the literature. Since the reduction of storage proteins is degraded by the offending action and exopeptidases, proteolytic enzymes convert the storage proteins into soluble peptides that are further hydrolyzed into free amino acids, which are then mobilized to the embryonic axis to support growth [46,47].

## 4. Materials and Methods

### 4.1. Site Description

The experiment was conducted in a protected environment at the experimental area of São Paulo State University (UNESP), in São Manuel—SP (22°46′ S, 48°34′ W and altitude of 740 m). During the experiment, the average maximum daily temperature was 28.7 °C, the minimum was 22.6 °C, and the maximum and minimum relative humidity were 73 and 54%, respectively. The experiment was conducted in pots (13 L); the soil used in the pots was fertilized and corrected with limestone as is recommended by Bulletin 100 [44], and the top dressing by fertigation according to ref. [48].

The chemical characteristics of the soil used were: pH (CaCl_2_): 4.4; organic matter: 5 g dm^−3^; P(resin): 2 mg dm^−3^; H+Al: 26 mmol_c_ dm^−3^; K: 1.1 mmol_c_ dm^−3^; Ca: 33 mmol_c_ dm^−3^; Mg: 4 mmol_c_ dm^−3^; sum of bases: 39 mmol_c_ dm^−3^; capacity of exchange cation: 64 mmol_c_ dm^−3^; base saturation: 60%.

### 4.2. Experiment Conduction and Experimental Design

Sowing was performed on 24th July 2017, and seedling transplantation at 47 days after sowing. An inbred line (SK 1730) from Sakata Seeds was used in this study. The management approach involved the withdrawal of sprouts until the appearance of the first flower, drip irrigation twice a day, and chemical pest and disease control when needed.

The experiment design was randomized in blocks, in a 4 × 2 factorial arrangement, and in four replications. The first factor comprised four maturation periods (35, 50, 65 and 80 days after anthesis (DAA)), and the second, the fruit post-harvest management (with and without the fruit rest for seven days after harvesting at laboratory conditions (25 ± 2 °C)). Ten plants were evaluated per plot, and all fruits fixed on the plants were harvested without thinning.

**Figure 3:**
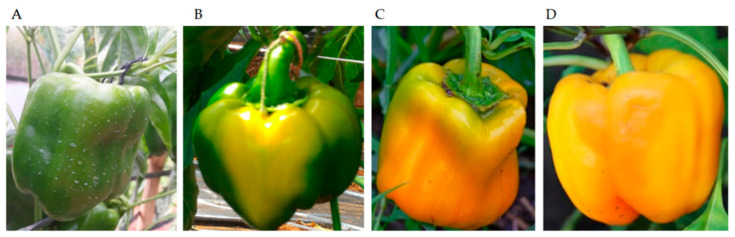
Visual aspects of sweet pepper fruit at 35 (**A**), 50 (**B**), 65 (**C**) and 80 (**D**) days after anthesis.

To determine the maturation period, all flowers were marked on the day of their anthesis. The harvests were performed when the fruits had the maturity stage corresponding to 35, 50, 65 and 80 DAA. Half of the fruits had their seeds extracted on the day of harvesting (without rest), and then half remained post-harvest, resting before seed extraction. At harvest time, the visual appearance of the fruits was: fully green fruits at 35 DAA; fruits with transient coloration from green to yellow at 50 DAA; fruits with 75% bright yellow color at 65 DAA; and at 80 DAA, the fruits were 100% yellow, but opaque and with less pulp firmness (Figure 3). Harvests were carried out manually, using scissors to separate the fruit from the mother plant.

### 4.3. Seed Analysis

Seed water content was determined immediately after fruit extraction by the oven method at 105 ± 3 °C for 24 h, using 10 g of seeds [46]. After extraction, the seeds were put in a dry chamber (40% relative humidity and 20 °C) to reduce the seed water content to approximately 8% for storage.

The seed characteristics evaluated were the dry weight of one thousand seeds (DWTS), macronutrients (N, P, K, Ca, Mg and S) and protein (albumin, globulin, prolamine and glutelin) content.

To determine the dry weight of one thousand seeds (DWTS), seeds were dried in a forced-air oven at 65 °C until they reached a constant weight and weighed on a scale of 0.0001 g [49].

Sulfuric digestion was used to obtain the extract in order to determine the content of N, while P, K, Ca, Mg and S content were extracted by nitroperchloric acid digestion and determined by atomic absorption spectrophotometry, as described by AOAC [50]. To determine the contents of albumin, globulin, glutelin and prolamine proteins, the methodology proposed by ref. [51] was used. For all determinations, four replications were used.

### 4.4. Data Analysis

The data were submitted for analysis of variance (F test) and, when significant, to compare the post-harvest resting periods of the fruits, the means were considered different by the F-test (*p* < 0.05). The effects of maturation periods were analyzed by regression analysis (*p* < 0.005).

## 5. Conclusions

The determination of sweet pepper seed quality is affected by the stage of maturation and the presence of rest for 7 days. This is mainly due to the reduction of K, Ca and Mg, essential macronutrients for good germination. However, the reserve protein content (globulin and prolamine) increased due to the presence of maturation.

In this way, obtaining a good seed production with post-harvest rest helps with the accumulation of calcium, albumin and glutelin. Therefore, harvesting the sweet pepper fruit and resting is a management strategy that can improve the quality of seeds.

## Figures and Tables

**Table 1:** Dry weight of one thousand seeds (DWTS), phosphorus (P), potassium (K), calcium (Ca) and magnesium (Mg) content in sweet pepper seeds as a function of post-harvest resting of fruits.

Post-Harvest Resting	DWTS	P	K	Ca	Mg
	g kg^−1^ of Dry Matter				
Without	4.3b	4.7a	12.4a	0.87b	2.4a
With	5.2a	4.4b	12.3a	1.02a	2.4a
CV (%)	2.5	1.41	4.07	8.79	7.10

Means followed by the same letter do not differ from each other by the F-test at 5% probability.

**Table 2:** Nitrogen (N) and sulfur (S) content of sweet pepper seeds without and with post-harvest resting of fruits at each maturation stage.

Post-Harvest Resting	N Content (g kg^−1^ of Dry Matter)				S Content (g kg^−1^ of Dry Matter)			
	Days after Anthesis							
	35	50	65	80	35	50	65	80
Without	30a	30a	29b	28b	1.9b	2.1a	2.0a	1.9a
With	28b	30a	31a	30a	2.1a	1.9b	1.8b	1.8a
CV (%)	3.65				6.43			

Means followed by the same letter in each column and for each nutrient do not differ by the F-test at 5% probability.

**Table 3:** Albumin, globulin, prolamine and glutelin content in sweet pepper seeds as a function of post-harvest resting of the fruits.

Post-Harvest Resting	Albumin	Globulin	Prolamine	Glutelin
	mg g^−1^ of Dry Matter			
Without	59.6b	17.5a	6.9a	13.5b
With	61.3a	16.7a	5.8b	16.2a
CV (%)	2.2	8.5	8.8	7.7

Means followed by the same letter do not differ from each other by the F-test at 5% probability.

## Data Availability

Not applicable.

## References

[1] Aguirre M., Kiegle E., Leo G., Ezquer I. (2018). Carbohydrate reserves and seed development: An overview. Plant Reprod..

[2] Bang T.C., Husted S., Laursen K.H., Persson D.P., Schjoerring J.K. (2021). The molecular–physiological functions of mineral macronutrients and their consequences for deficiency symptoms in plants. New Phytol..

[3] Ikram A., Saeed F., Afzaal M., Imran A., Niaz B., Tufail T., Hussain M., Anjum F.M. (2021). Nutritional and end-use perspectives of sprouted grains: A comprehensive review. Food Sci. Nutr..

[4] Kołton A., Wojciechowska R., Leja M. (2012). The effect of various light conditions and different nitrogen forms on nitrogen metabolism in pepper fruits. Folia Hortic..

[5] Zhang J., Lv J., Dawuda M.M., Xie J., Yu J., Li J., Zhang X., Tang C., Wang C., Gan Y. (2019). Appropriate ammonium-nitrate ratio improves nutrient accumulation and fruit quality in pepper (*Capsicum annuum* L.). Agronomy.

[6] Mouzo D., Bernal J., López-Pedrouso M., Franco D., Zapata C. (2018). Advances in the biology of seed and vegetative storage proteins based on two-dimensional electrophoresis coupled to mass spectrometry. Molecules.

[7] Shewry P.R., Napier J.A., Tatham A.S. (1995). Seed storage proteins: Structures and biosynthesis. Plant Cell.

[8] Tan-Wilson A.L., Wilson K.A. (2012). Mobilization of seed protein reserves. Physiol. Plant..

[9] Koornneef M., Bentsink L., Hilhorst H. (2002). Seed dormancy and germination. Curr. Opin. Plant Biol..

[10] Han C., Yang P. (2015). Studies on the molecular mechanisms of seed germination. Proteomics.

[11] Nakada-Freitas P.G., Lanna N.B.L., Silva P.N.L., Bardiviesso E.M., Tavares A.E.B., Claudio M.T.R., Cardoso A.I.I., Magro F.O., Araujo H.S. (2020). The physiological quality of “chilli pepper” seeds, extracted from fruits harvested at different stages of maturation, with and without post-harvest rest. Aust. J. Crop Sci..

[12] Colombari L.F., da Silva G.F., Chamma L., Chaves P.P.N., Martins B.N.M., Jorge L.G., de Lima Silva P.N., Putti F.F., Cardoso A.I.I. (2021). Maturation and resting of sweet pepper fruits on physiological quality and biochemical response of seeds. Brazilian Arch. Biol. Technol..

[13] Medeiros A.D., Zavala-León M.J., da Silva L.J., Oliveira A.M.S., dos Santos Dias D.C.F. (2020). Relationship between internal morphology and physiological quality of pepper seeds during fruit maturation and storage. Agron. J..

[14] Ohanenye I.C., Tsopmo A., Ejike C.E.C.C., Udenigwe C.C. (2020). Germination as a bioprocess for enhancing the quality and nutritional prospects of legume proteins. Trends Food Sci. Technol..

[15] Cisternas-Jamet J., Salvatierra-Martínez R., Vega-Gálvez A., Stoll A., Uribe E., Goñi M.G. (2020). Biochemical composition as a function of fruit maturity stage of bell pepper (*Capsicum annum*) inoculated with Bacillus amyloliquefaciens. Sci. Hortic..

[16] Yildirim K.C., Canik Orel D., Okyay H., Gursan M.M., Demir I. (2021). Quality of immature and mature pepper (*Capsicum annuum* L.) seeds in relation to bio-priming with endophytic pseudomonas and *Bacillus* spp.. Horticulturae.

[17] Bewley J.D., Bradford K.J., Hilhorst H.W.M., Nonogaki H. (2013). Seeds.

[18] Wolny E., Betekhtin A., Rojek M., Braszewska-Zalewska A., Lusinska J., Hasterok R. (2018). Germination and the early stages of seedling development in *Brachypodium distachyon*. Int. J. Mol. Sci..

[19] Linkies A., Leubner-Metzger G. (2012). Beyond gibberellins and abscisic acid: How ethylene and jasmonates control seed germination. Plant Cell Rep..

[20] Yan D., Duermeyer L., Leoveanu C., Nambara E. (2014). The functions of the endosperm during seed germination. Plant Cell Physiol..

[21] Bortey H.M., Dzomeku B.M. (2016). Fruit and seed quality of okra [*Abelmoschus esculentus* (L.) Moench] as influenced by harvesting stage and drying method. Indian J. Agric. Res..

[22] Singkaew J., Miyagawa S., Wongs-Aree C., Vichitsoonthonkul T., Sokaokha S., Photchanachai S. (2017). Season, fruit maturity, and storage affect on the physiological quality of F1 hybrid ‘VTM580’ tomato seeds and seedlings. Hortic. J..

[23] Lima J.M.E., Smiderle O.J. (2014). Physiologic quality of pepper seeds obtained from to fruit maturation and storage and storage. Semin. Ciências Agrárias.

[24] Vidigalde Souza Vidigal D., Dias D.C.F.S., de Rezende Von Pinho E.V., dos Santos Dias L.A. (2009). Physiological and enzymatic changes during pepper seeds (*Capsicum annuum* L.) maturation. Rev. Bras. Sementes.

[25] Caixeta F., Von Pinho É.V.R., Catão R.M.G., Pereira P.H.A.R., Catão H.C.R.M. (2014). Physiological and biochemical alterations during germination and storage of habanero pepper seeds. African J. Agric. Res..

[26] Nogueira J.L., da Silva B.A., Mógor Á.F., de Souza Grzybowski C.R., Panobianco M. (2017). Quality of organically produced bell pepper seeds. J. Seed Sci..

[27] Pinheiro D.T., de Oliveira R.M., de Souza Silveira A., León M.J.Z., Brum L.B.T.L., dos Santos Dias D.C.F. (2020). Antioxidant enzyme activity and physiological potential of *Capsicum baccatum* var. baccatum seeds as a function of post-harvest storage of fruit. J. Seed Sci..

[28] de Souza Vidigal D., dos Santos Dias D.C.F., dos Santos Dias L.A., Finger F.L. (2011). Changes in seed quality during fruit maturation of sweet pepper. Sci. Agric..

[29] Kano C., Cardoso A.I.I., Villas Bôas R.L. (2010). Macronutrient content in lettuce affected by potassium side dressing. Hortic. Bras..

[30] Cardoso A.I., Claudio M.T., Nakada-Freitas P.G., Magro F.O., Tavares A.E. (2016). Phosphate fertilization over the accumulation of macronutrients in cauliflower seed production. Hortic. Bras..

[31] Masclaux-Daubresse C., Daniel-Vedele F., Dechorgnat J., Chardon F., Gaufichon L., Suzuki A. (2010). Nitrogen uptake, assimilation and remobilization in plants: Challenges for sustainable and productive agriculture. Ann. Bot..

[32] Sandhu N., Sethi M., Kumar A., Dang D., Singh J., Chhuneja P. (2021). Biochemical and genetic approaches improving nitrogen use efficiency in cereal crops: A review. Front. Plant Sci..

[33] Doyle J.W., Nambeesan S.U., Malladi A. (2021). Physiology of nitrogen and calcium nutrition in blueberry (*Vaccinium* sp.). Agronomy.

[34] Marschner P. (2012). Mineral Nutrition of Higher Plants.

[35] Schachtman D.P., Reid R.J., Ayling S.M. (1998). Phosphorus uptake by plants: From soil to cell. Plant Physiol..

[36] White P.J., Veneklaas E.J. (2012). Nature and nurture: The importance of seed phosphorus content. Plant Soil.

[37] Görlach B.M., Sagervanshi A., Henningsen J.N., Pitann B., Mühling K.H. (2021). Uptake, subcellular distribution, and translocation of foliar-applied phosphorus: Short-term effects on ion relations in deficient young maize plants. Plant Physiol. Biochem..

[38] Lemaire G., Sinclair T., Sadras V., Bélanger G. (2019). Allometric approach to crop nutrition and implications for crop diagnosis and phenotyping. A review. Agron. Sustain. Dev..

[39] Sawan Z.M., Fahmy A.H., Yousef S.E. (2011). Effect of potassium, zinc and phosphorus on seed yield, seed viability and seedling vigor of cotton (*Gossypium barbadense* L.). Arch. Agron. Soil Sci..

[40] Ceylan Y., Kutman U.B., Mengutay M., Cakmak I. (2016). Magnesium applications to growth medium and foliage affect the starch distribution, increase the grain size and improve the seed germination in wheat. Plant Soil.

[41] Chandra N., Pandey N. (2016). Role of sulfur nutrition in plant and seed metabolism of *Glycine max* L.. J. Plant Nutr..

[42] Mondal S., Pramanik K., Panda D., Dutta D., Karmakar S., Bose B. (2022). Sulfur in Seeds: An Overview. Plants.

[43] Dos Reis A.R., Boleta E.H.M., Alves C.Z., Cotrim M.F., Barbosa J.Z., Silva V.M., Porto R.L., Lanza M.G.D.B., Lavres J., Gomes M.H.F. (2020). Selenium toxicity in upland field-grown rice: Seed physiology responses and nutrient distribution using the μ-XRF technique. Ecotoxicol. Environ. Saf..

[44] Ramakrishna V. (2007). Mobilization of albumins and globulins during germination of Indian bean (*Dolichos lablab* L. var. lignosus) seeds. Acta Bot. Croat..

[45] Zhao M., Zhang H., Yan H., Qiu L., Baskin C.C. (2018). Mobilization and role of starch, protein, and fat reserves during seed germination of six wild grassland species. Front. Plant Sci..

[46] Schlereth A., Standhardt D., Mock H.-P., Müntz K. (2001). Stored cysteine proteinases start globulin mobilization in protein bodies of embryonic axes and cotyledons during vetch (*Vicia sativa* L.) seed germination. Planta.

[47] Müntz K., Belozersky M.A., Dunaevsky Y.E., Schlereth A., Tiedemann J. (2001). Stored proteinases and the initiation of storage protein mobilization in seeds during germination and seedling growth. J. Exp. Bot..

[48] Trani P.E., Tivelli S.W., Carrijo O.A. (2011). Fertirrigação em Hortaliças.

[49] Brasil (2009). Regras Para Análise de Sementes.

[50] AOAC (2016). Official Methods of Analysis of AOAC International.

[51] Bradford M. (1976). A Rapid and sensitive method for the quantitation of microgram quantities of protein utilizing the principle of protein-dye binding. Anal. Biochem..

